# Supplementary figures and images for: Gut Microbial Dysbiosis Associated with Type 2 Diabetes Aggravates Acute Ischemic Stroke
Source: mSystems. 2021 Dec 21;6(6):e01304-21. doi: 10.1128/msystems.01304-21 (PMC8693450; doi:10.1128/msystems.01304-21)

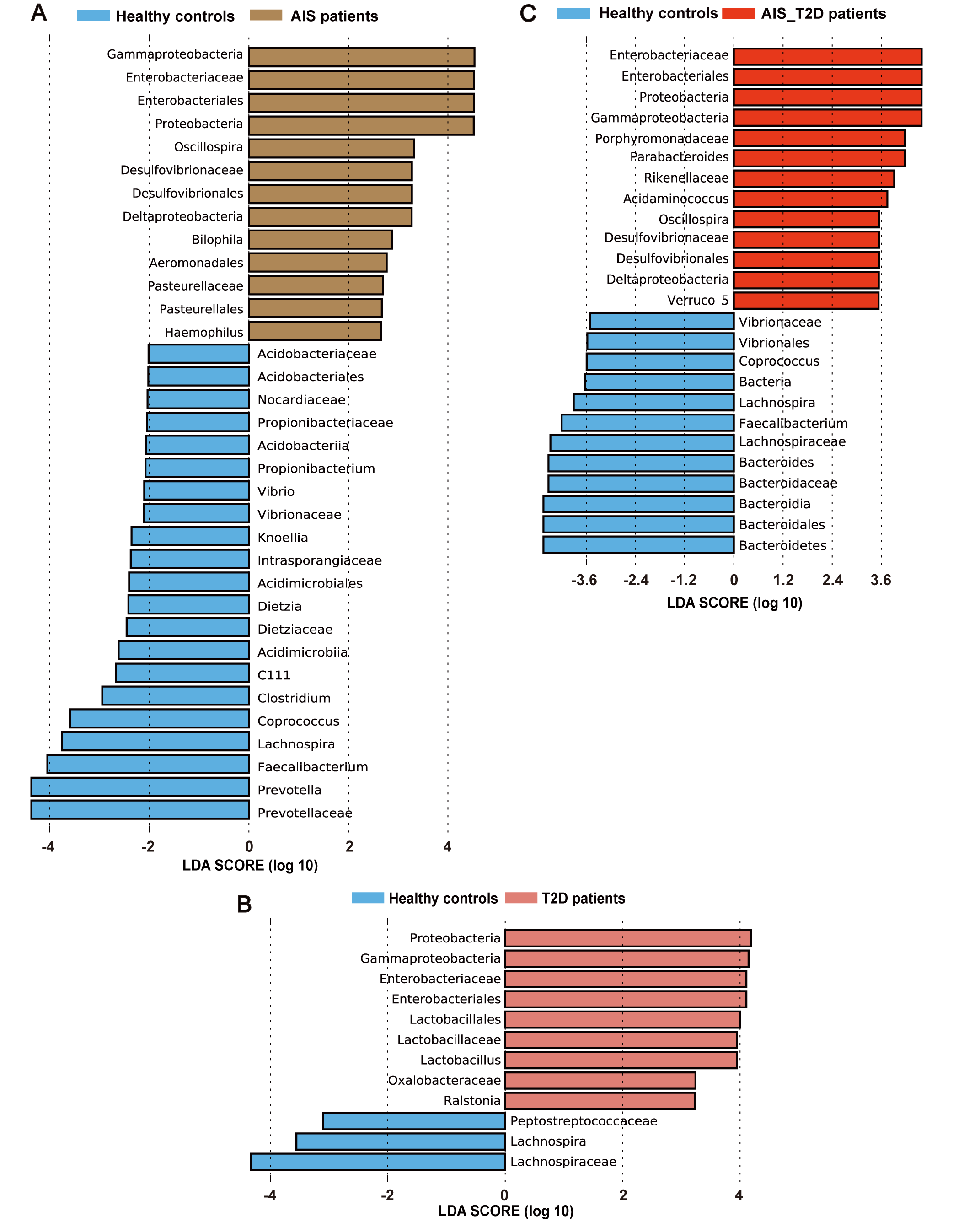

Supplement: FIG S1 [file msystems.01304-21-sf001.tif]

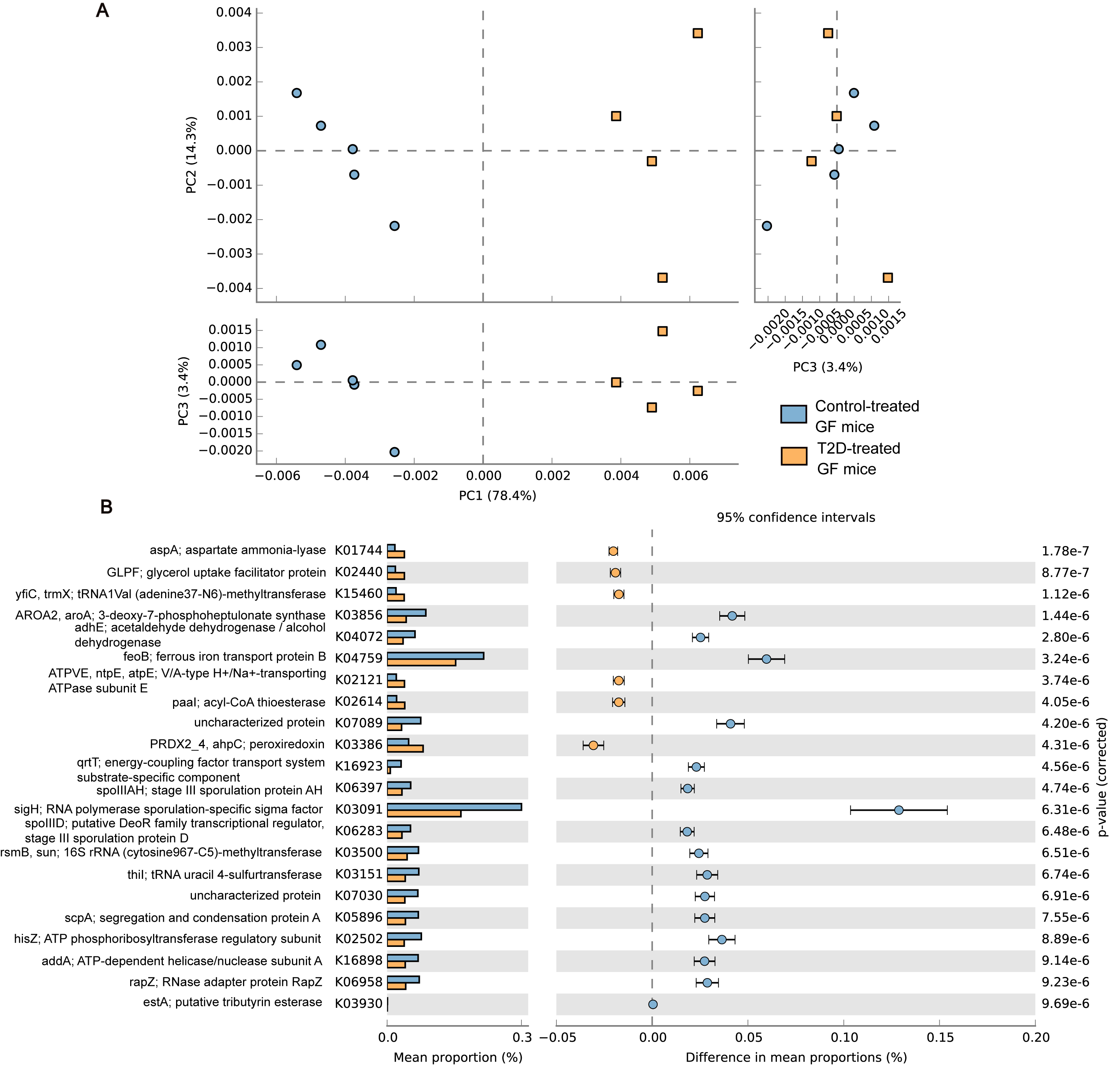

Supplement: FIG S2 [file msystems.01304-21-sf002.tif]
